# Report of the Committee on Dental Education

**Published:** 1870-11

**Authors:** M. S. Dean, J. N. Crouse


DENTAL REGISTER.

VOL. XXIV.] NOVEMBER, 1870. [No. 11.

### Original Communications.

### REPORT OF THE COMMITTEE ON DENTAL EDUCATION.

BY M. S. DEAN AND J. N. CROUSE.

MR. PRESIDENT: In the report of last year your committee dwelt upon the importance and necessity of a *good preliminary education* for the Dental student, and urged the Association to demand in future a thorough English education at least of these pupils before admitting them as students of Dentistry.

Your present committee would emphatically reiterate this, believing that no amount of professional study can fully compensate for the want of it, and that our specialty can never attain an honorable position among the professions until its members rigidly insist upon this essential prerequisite.

With this brief reference to preliminary education, your committee will proceed to the subject of office and college instruction, in regard to which, in some respects, there is considerable diversity of opinion, even among the leading members of our profession. With all due deference to those whose opinions may differ somewhat from their own, your committee will point out what they consider some of the faults in the present system of Dental education, and endeavor to suggest some improvements in the course of instruction to be pursued by the student.

It is a well established truth that theory alone will never make a man successful in any department of active life. He may be thoroughly acquainted with all the facts relating to his special avocation, yet fail entirely as a business or professional man. To attain success he must combine practice with theory. This seems to be a general law, and in accordance with it your committee will endeavor to call more earnest attention to the subject of office instruction, believing that the student should be tought not only the theoretical principles of Dentistry, which are so imperfectly laid down in many of our text books, but more especially their application as demanded at the chair and in the laboratory. In the preceding reports upon Dental education, very little was said about office instruction. It has either been regarded as a relic of former years, or as unworthy of notice compared with collegiate education. But to your committee it seems vastly important that the student should receive, in addition to a full course of collegiate education, thorough and systematic instruction in the different departments of study, and in the *practice* of Dentistry, in the office of a competent and conscientious practitioner.

But just how long the pupil should study in a Dental office, or just how far he should be advanced in the different branches of study before it is advisable for him to attemd a Dental college, is a matter difficult to determine. Yet, in the opinion of your committee, he should receive his first course of lectures as early as possible, or at least as soon as he is sufficiently acquainted with the different branches taught, to understand and fully comprehend the lecturer; although this session might be less laborious to him and more agreeable to the professors, had he more thoroughly mastered the fundamental branches, especially anatomy, physiology, and chemistry, before entering his first collegiate course.

By taking this course early, through the guiding influence of professional instructors, he will be more apt to start right and to keep on in the right direction; and on his return to the office of his preceptor, he will be more certain to pursue a thorough, systematic course of study than he would otherwise do. For the Dental college, if it is what it should be, has an inspiring influence upon the student, and stimulates him to generous emulation in his class, and makes him ambitious and zealous to excel in his professional studies.

Of laboratory instruction, but little need be said. Since the introduction of rubber for artificial plates, Dentists generally have regarded this style of work as so important to their students that they have kept them taking impressions, making casts, vulcanizing, etc., until the poor pupil has come to consider this mechanical drudgery the scientific part of Dentistry. Of course the preceptor has not been at all selfish in this matter. The highest good of the student has been his controlling motive. He realizes that if the student would be successful, he must be enthusiastic, and as the surest way of kindling his enthusiasm, he places him week after week and month after month amidst the very attractive and inspiring surroundings of the work shop! Now without being understood as detracting from the value in the least of a proper laboratory instruction, would it not be better for the student, instead of consuming so much of his time with these inferior manipulations, that he receive more of his preceptor's time in teaching him the more difficult parts of plate work, selecting and arranging teeth in a skillful and artistic manner, and the working of the different metals? And yet the policy of placing laboratory instruction, so prominent as to overshadow the most important branch of Dentistry—the operative—is decidedly objectionable.

Too much can not be said in favor of thorough instruction in the operative department. Daily practice at the chair is of the utmost importance to the student's after success. He should procure his own patients and perform his own operations under the strict directions of his preceptor. Close attention and criticism on the part of his preceptor are invaluable to the student while in this department of study. Let his work be progressive, from simple to the more complicated and difficult. The student may thus be enabled to see the substantial progress he is daily making, inciting him to renewed efforts and diligence. No defects should be allowed to pass unnoticed. Neatness and precision should be inculcated in such a manner as not to discourage the student, who sees so much at this point to learn.

If it is objected that the practitioner can not spend so much time with his student as would be required by this method of instruction, it may be answered, he should receive a proper compensation for his services. To this reply the objector may say that poor young men could not enter the profession if an adequate fee were required for instruction. It may again be answered, the Dental profession is not an asylum for paupers. While every reasonable favor should be extended to worthy young men, our profession should not be degraded to accommodate those who are too indolent to acquire the means necessary to pursue the course indicated. By raising the standard of preparation, and asking a just compensation for instruction, a better class of men will join our ranks. Young men without means, if possessed of sufficient talent and ambition to prove an acquisition to our number, will find ways of securing the funds necessary to prosecute their studies. Our colleges will have fewer dunces, and will graduate better Dentists. The people will be more satisfactorily served, and our noble occupation will take a higher position among the professions.

But before the profession reaches this elevated position, our schools of instruction, their advantages for a complete Dental education, their facilities for learning, must likewise be improved and increased.

The number of reputable Dental colleges now in active operation is nine, while the list of graduates emanating from these is not more than three or four for each professor. This state of affairs would seem to show, in the opinion of your committee, that there are a greater number of Dental colleges than are needful or profitable; that there is too great a disproportion between the number of these institutions and the students; in fact, that there are too many of them that are so feebly supported that it must be impossible for them to furnish adequate facilities and the very best qualified instructors. Most of these colleges are certainly supported to a considerable extent by the teachers, instead of the teachers being supported by the colleges. And while this exhibits a noble and self-sacrificing spirit on the part of the professors, the question arises, are they serving the best interests of the profession? Are they really a benefit to the Dental student? Not, are Dental colleges promoting the interests of the profession and benefiting the student? for this no one will deny. But would the interests of the student be better served if there were *a less* number of Dental colleges? Your committee are of the opinion that they would. For although it can not be denied that some of the very best talent is now employed in our colleges that can be found in the profession, it can not, on the other hand, be denied that the teacher who gives his services gratuitously, who takes the time from his office business that he employs in the preparation of his lectures, who devotes the time to teaching which is absolutely needed for the relaxation of the mind and the recreation of the body. It can not be denied, we say, that the teacher who works without compensation, with limited facilities and appliances for instruction, and with a meager class to instruct, must fail to be as useful and successful a teacher as if he was fully compensated for his services, and had ample time and every needful facility for instruction. But a complete corps of such professors can not be employed in our colleges until the number of pupils is greatly increased, or the number of colleges is greatly diminished; and until that time shall come the Dental student can not acquire that complete professional education which should be obtained for the amount of time and means bestowed upon it.

While it is true there are some who may be found willing to work for the good of the profession, who may teach for the love or the honor which the avocation affords, making up in zeal, perhaps, for what they lack in proper scientific preparation for teaching, yet their labors can only be of short duration. Teachers can not live a long time upon the honor which a professorship in a Dental college affords. This sort of diet, though pleasant to the taste, is not sufficiently nutritious to sustain the most ambitious. The stability of our colleges must depend upon a more secure and substantial foundation.

By establishing a few educational centers in favorable localities, instead of planting a Dental college upon every hill-top—or in every flourishing city, with an adequate staff of capable professors, men of enlarged views and eminent for learning, possessed not only of the highest mental and moral qualities, but also endowed with the *gift* and familiar with the science of teaching, and, supplying these schools with every possible facility for instruction, the student may be enabled to receive a far better Dental education with no increase of time or expense, and our colleges would be more prosperous and self-supporting institutions. Then the title of D. D. S. would be an honor to its possessor, and its possessor would be worthy of the honorable title.

But it may be said the standard of *Dental* colleges here presented rises far above the present position of the best medical colleges in the land, which is true. But we need not entertain any fears of surpassing these in education, even if we do in usefulness and skill; for the medical colleges will also make the science of medicine both easier to attain and more worthy of attainment than it now is, although the facilities for the education of the medical student are now much greater than are those of the student of Dentistry. They suffer far less from the want of text books adapted to their use, and their faculties generally are more thoroughly educated. We shall have to raise the character of our Dental institutions not a little before they stand on a level with the medical. This must and will be done, and unless the present onward course be checked, will soon be accomplished.

But to accomplish this our office and collegiate instruction not only needs improvement, but our text books also, for these are sadly deficient and defective. Yet in the fundamental branches of study we stand equal to the medical profession, for in *these* we borrow our text books from it. We have also works on operative and mechanical Dentistry that come up closely to the times; but aside from these we have no text books which treat of the special branches of our profession that are really worth the name. On physiology, pathology and therapeutics, and even oral surgery, there are no standard text books worthy of being placed in the hand of the student. He must take those which are prepared especially for the student of medicine, which however will answer every purpose until he wishes to apply their principles in his special practice. The student suffers more from the want of complete systematic text books that are adapted especially to his wants, than from any other cause. With such as are furnished him he must work to a great disadvantage. He must grapple the science at arm's length and waste much of his strength and energies.

In our common schools vast improvement is constantly being made in the text books and the methods of teaching. But how is it with the professional student? Save in our own specialty, he is plodding along over the same old road that the ancients trod, and which in some places has been but little improved since the days of Æscalapius. The same old obstacles lie in the path that have been there for the last century, and only when worn down by the feet of these disciples, are as difficult to climb as ever. Our profession is really the most live, progressive profession of the present age. And our colleges, too, are doing as much in pulling it along, in bending and inclining the professional twig, or more perhaps than all the other agencies put together; yet we must not allow them to halt in their glorious march for a moment, but encourage them to advance as rapidly as possible. For every improvement they make in their system of education we must demand of them two more. Shall we then, it may be asked, require them to give the student in their colleges a full medical course of instruction in addition to that in Dentistry? Your committee would answer emphatically, yes. Yes, when these two professions may be perfectly mastered in the time which must now be devoted to one. Yes, when the plan of instruction has been perfected to such a degree that they can be taught as one harmonious whole. When the medical and Dental text books are so perfected and arranged as to blend together, each part adapted to the others making a complete unity. Yes, when this medical education will not be acquired at the expense or neglect of a most thorough course of Dentistry. Then, and not till then, should we require a full course of medical instruction as a part of our professional education.

Your committee are of the opinion that the Dental profession should not demand of their pupils a higher degree of professional education than is required of the general practitioner of medicine Nor should his term of pupilage greatly exceed that of the medical student, which it certainly must do if he become doctor of both. And as the practice of these professions should be kept distinct and separate, his professional studies need not *necessarily* embrace only those branches of medicine which shall add to his qualifications as a Dentist. This being the case it only remains to be determined what particular branches and what proportion of these branches should be embraced in the course of Dental study.

If we can acquire only a certain amount of knowledge, if we must be limited in our acquisitions, we should obtain that which would prove the most available and the most useful in our daily avocations in life. If students have the means and the capacity to become a Doctor of Medicine and of Dental Surgery, so much the better. To such great honor and respect is due. But such medical colleges as may confer the degree of Doctor of Dental Medicine or Surgery, in which Dentistry is incidentally and inadequately taught as the collateral, and not the main department of education, should be shunned by every Dental student and denounced by the whole Dental profession. For such institutions, in adding a Dental professor to their faculty, only use him as a decoy to increase the number of their students. These remarks do not apply to those medical colleges which may, and very properly, too, teach the elements of Dentistry, but to such as may award Dental diplomas without giving a complete course of Dental education.

But just what should constitute the course of study which would make the best practical Dentists, and at the same time store his mind with such a knowledge of the sciences as to make his labors a pleasure instead of a monotonous routine, your committee will not attempt to definitely determine. There is no doubt, however, that general anatomy, physiology, pathology and chemistry, shall be the fundamental studies of the Dental student, and that these should be thoroughly mastered by him. Then he should be supplied with *improved* text books, specially pertaining to the teeth and adjacent parts, on anatomy, physiology, surgery, chemistry, therapeutics and histology, besides those on operative and mechanical Dentistry. Here we have a range of study that comprehends and embraces all the student can acquire in a three years pupilage, and which would require more time than is usually given to medicine or law.

But the time must come, and is not far distant, when the Dental student, aided by the improved machinery of instruction, will go over a more extended field than has been here laid out for him, and reap a more abundant harvest of professional knowledge.

But while the young profession is advancing as rapidly as it is at present, the *Dental journals* and *Dental associations* must act a prominent part especially in *perfecting* the education of the

Dentist. Unless the graduate in Dentistry pay close attention to the Dental journals which contain the monthly fashion reports, and carefully note the changes in style that are constantly being made, and unless he attend the Dental associations, where the different modes are discussed, and where each member assists the other in remodeling his professional toga, he will soon find the elegant robe he wore so proudly from his *alma mater* out of fashion when compared with the newer ones of the next class that succeeds him. These magazines and societies are indispensable sources of instruction to the collegiate as well as to the practitioner, who may have been less fortunate in his earlier professional education.

In conclusion, when the Dental student is required to possess at least a good English education before entering upon the study of Dentistry, and when he receives such office instruction as a conscientious educated practitioner will be likely to give him—when the text books are better adapted to his special wants, and when the number of our colleges are in reasonable proportion to the pupils taught, and the faculties are selected from the most accomplished teachers that can be found—men of the highest mental culture and soundest moral qualities, and withal adapted by nature as well as education to the science of teaching; when the professors are as liberally supported as are men of marked ability in other professions and callings, and when our colleges are furnished with every appliance that can aid the student in acquiring the most knowledge in the shortest time and in the easiest way. Then our specialty will be composed of professional men in the true sense of the term, and will not be obliged to *assert* its claim to the title of a liberal profession; for this honor will be freely bestowed on it. Then the graduates will go forth as the coin from the mint, with their true professional value stamped upon them, and bearing such a moral impress of the institution from which they emanate that it will not be effaced in professional strife in after years.

